# Tracheobronchopathia osteochondroplastica: clinical, bronchoscopic, and comorbid features in a case series

**DOI:** 10.1186/s12890-022-02225-2

**Published:** 2022-11-17

**Authors:** Antoine Dumazet, Claire Launois, Francois Lebargy, Romain Kessler, Hervé Vallerand, Pierre Schmitt, Christophe Hermant, Sandra Dury, Maxime Dewolf, Julien Dutilh, Maher Abouda, Marion Ferreira, Ihab Atallah, Samy Lachkar, Jérémy Charriot, Stéphane Jouneau, Yurdagul Uzunhan, Stéphane Chouabe, Benjamin Coiffard, Hervé Dutau, Jean Hagenburg, Amandine Briault, Valérian Dormoy, Marion Lirsac, Jean-Michel Vergnon, Gaetan Deslee, Jeanne-Marie Perotin

**Affiliations:** 1 Department of Respiratory Diseases, University Hospital of Reims, 45 rue Cognacq Jay, 51100 Reims, France; 2 Pulmonology Department, University Hospital, Strasbourg, France; 3 Sarreguemines, France; 4 Pulmonology Department, Larrey University Hospital, Toulouse, France; 5 Pulmonology Department, University Hospital, Poitiers, France; 6 Faculty of Medicine of Tunis, F.S.I. Hospital, University of Tunis El-Manar, 2070 Tunis, TN Tunisia; 7 Department of Pneumology and Respiratory Functional Exploration, University Hospital of Tours, Tours, France; 8 Otolaryngology Department, Grenoble Alpes University Hospital, Grenoble, France; 9 Department of Pulmonology and CIC-CRB 1404, Rouen University Hospital, Rouen, France; 10 Department of Respiratory Diseases, University Montpellier, CHU Montpellier, Montpellier, France; 11 Department of Pulmonology, Rennes University Hospital, Competence Center for Rare Pulmonary Diseases, IRSET UMR 1085, Rennes 1 University, 35033 Rennes, France; 12 Department of Pneumology, Assistance Publique-Hôpitaux de Paris, Avicenne Hospital, INSERM UMR 1272, Sorbonne Paris Nord University, Bobigny, France; 13 Department of Respiratory Diseases, Charleville-Mézières, France; 14 Department of Respiratory Medicine and Lung Transplantation, Hôpital Nord, Marseille, France; 15 Department of Pulmonology, Grenoble Alpes University Hospital, Grenoble, France; 16 Inserm UMR-S 1250, University Hospital, Reims, France; 17 Department of Pathology, Hôpital Maison-Blanche, Reims, France; 18 Department of Chest Diseases and Thoracic Oncology, Hôpital Nord, CHU de Saint-Étienne, Saint-Étienne, France

**Keywords:** Tracheobronchopathia osteochondroplastica, Bronchoscopy, Tracheal stenosis, Case report

## Abstract

**Background:**

Tracheobronchopathia osteochondroplastica (TO) is a rare condition of unknown etiology. TO is characterized by submucosal nodules, with or without calcifications, protruding in the anterolateral walls of the trachea and proximal bronchi. The objective of this study was to describe TO features and associated comorbidities in a series of patients.

**Methods:**

Patients suffering from TO were retrospectively included by investigators from the Groupe d’Endoscopie Thoracique et Interventionnelle Francophone (GETIF). Demographic, clinical, comorbidities, bronchoscopic, functional, and radiological characteristics, and outcomes were recorded and analyzed.

**Results:**

Thirty-six patients were included (69% male with a mean of 65 ± 12 years). Chronic symptoms were described by 81% of patients including cough (74%) and dyspnea on exertion (74%). TO was associated with COPD in 19% of the cases and gastroesophageal reflux disease in 6%. A mild to severe airflow obstruction was present in 55% of the cases. CT scan showed tracheal submucosal nodules in 93% of patients and tracheal stenosis in 17%. Bronchoscopy identified TO lesions in the trachea in 65% of the cases, and 66% of them were scattered. A bronchoscopic reevaluation was performed in 7 cases, 9 ± 14 months [1–56] after initial diagnosis, and showed the stability of lesions in all cases. Three patients underwent interventional bronchoscopic treatment.

**Conclusion:**

The diagnosis of TO relies on typical bronchoscopic findings and can be evoked on a CT scan. Histologic diagnosis can be useful in atypical cases for differential diagnosis. Given its low consequences in terms of symptoms, lung functions, and evolution, no treatment is usually required.

## Background

Tracheobronchopathia osteochondroplastica (TO) is a rare condition of unknown etiology characterized by submucosal nodules with or without calcifications protruding in the anterolateral walls of the trachea and proximal bronchi [1]. The prevalence of TO has been estimated between 0.01 and 0.80%, 0.05% in a recent Chinese cohort [1]. This prevalence is probably underestimated partly because of non-specific symptoms and potential bronchoscopic misdiagnosis. The main differential diagnoses to consider are tracheobronchial papillomatosis, amyloidosis, sarcoidosis, and relapsing polychondritis [2].

Since its first description more than a century ago [3], about 500 cases of TO have been published as observations or small series [1, 4–6]. No guidelines are currently available for its diagnosis and therapeutic management. The objective of this study was to describe the clinical, functional, and morphological features of a cohort of patients with TO and to evaluate associated comorbidities.

## Methods

### Study design and methods

This multicenter observational study was conducted between November 2017 and November 2019 in 14 French and Tunisian university centers involved in interventional bronchoscopy and members of the Groupe d’Endoscopie Thoracique et Interventionnelle Francophone (GETIF): Bobigny, Charleville-Mézières, Grenoble, Marseille, Montpellier, Poitiers, Reims, Rennes, Rouen, Sarreguemines, Strasbourg, Toulouse, Tours, and Tunis. All patients had a diagnosis of TO from typical bronchoscopic and radiological features and/or histological findings. Histological analysis was therefore not mandatory for the diagnosis of TO to be confirmed and for patients to be included in the study. All methods were carried out per ethical guidelines and regulations. Informed consent was obtained from all participants included in the study. The study protocol was approved by the Institutional Review Board of the French learned Society for Respiratory Medicine – Société de Pneumologie de Langue Francaise) (CEPRO 2018–011).

A standard form was used to retrospectively record from patients’ files the details of patients and TO characteristics, including demographics, smoking status, occupational exposure, medical history, respiratory symptoms, pulmonary function tests (PFT) results, and radiological and histological findings. Bronchoscopic data included TO location, tracheal stenosis, and classification depending on nodules concentration in the mucosa: scattered, diffuse or confluent [6]. When available, microbiologic results of bronchial aspiration conventional culture were recorded. Therapeutic management and outcomes were recorded. Two authors (A.D and J.M.P) had full access to the data and take responsibility for the integrity of the data and the accuracy of the data analysis.

### Statistical analysis

All analyses were performed using Epi Info version 7.2. Quantitative variables are described as mean ± standard deviation and categorical variables in relative and absolute frequency.

## Results

### Patients

Thirty-six patients were included, five of them being previously reported [7–9]. They were mean aged 65 ± 12 years (36 to 88), and 20 (56%) were smokers (Table 1). Seven patients (19%) were asymptomatic. Respiratory symptoms were chronic and non-specific including chronic cough (*n* = 18, 75%) and dyspnea on exertion (*n* = 17, 71%). Mild hemoptysis was described in one case and acute lower respiratory tract infection in 7 cases (19%). In the 29 patients with respiratory symptoms, symptoms appeared between 0 and 168 months (mean 51 ± 49 months) before TO diagnosis.

**Table 1:** Demographic and clinical data

Characteristics		%
Numbers of cases	36	
Male	25	
Age (years)	65 ± 12	
Smokers	20	56
current smokers	4	21
ex-smokers	16	79
smoking history (pack-years)	36 ± 27	
Symptoms	29	81
acute	8	28
chronic	24	83
cough	18	75
exertional dyspnea	17	71
Pulmonary functional tests	27	75
FEV_1_/FVC < 0.7	15	55
FEV_1_ > 80%	3	11
FEV_1_ 50–80%	7	26
FEV_1_ < 50%	5	18
TLC < 80% of predicted value	0	0
FEV_1_, % of predicted value	82 ± 33	
TLC, % of predicted value	103 ± 29	

Data are numbers and percentages or mean ± standard deviation

*FEV*_*1*_ forced expiratory volume in the first second, *TLC* total lung capacity, *FVC* forced vital capacity

Analysis of patients’ environment identified occupational exposure in 20 patients (56%), including asbestos (*n* = 5), construction and public works (*n* = 4), textile industry (*n* = 3), and steel foundry (*n* = 3).

Comorbidities included COPD (*n* = 8, 19%), gastroesophageal reflux disease (GERD) (*n* = 5, 14%), chronic rhinosinusitis (*n* = 4, 11%) and tuberculosis (*n* = 2, 6%). No family history of TO was reported. PFT results were available for 26 patients and revealed airflow obstruction in 15 patients (55%) (Table 1).

### Radiological and histological findings

**Fig. 1:**
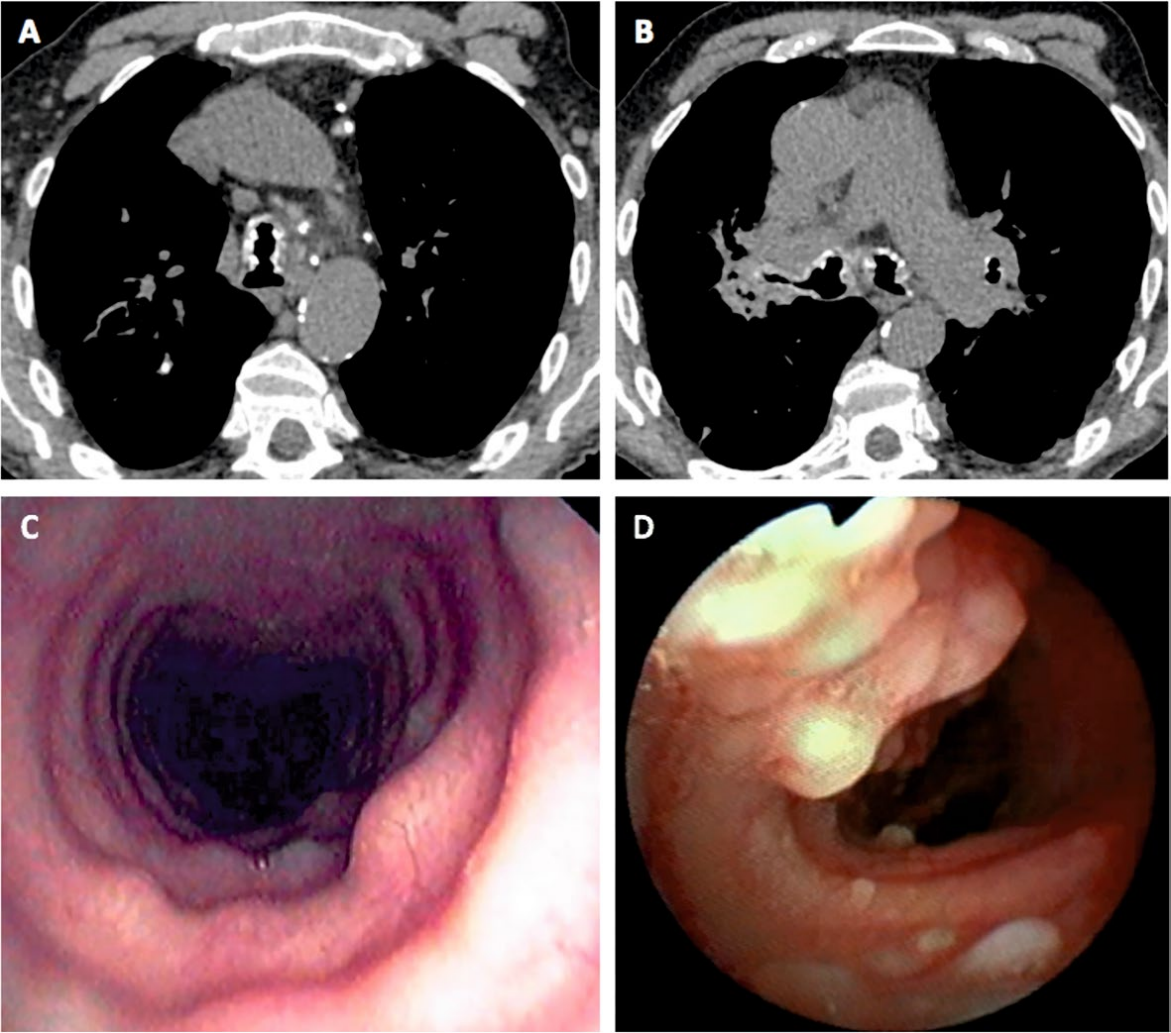
Radiologic and bronchoscopic features of tracheobronchopathia osteochrondroplastica. Chest CT scan shows submucosal nodules and calcifications in the trachea lumen and the proximal bronchi (**A**, **B**). Bronchoscopy reveals submucosal scattered (**C**) or diffuse (**D**) nodules protruding in the lumen of the trachea

Thoracic imaging results (chest X-ray and/or CT scan) were available for 32 patients. Chest X-Ray was described as normal (*n* = 13/25, 52%) or exhibited tracheal calcifications (*n* = 5, 20%), tracheal stenosis (*n* = 1) and tuberculous sequelae associated with alveolar consolidation (*n* = 1). The patterns of CT-scan tracheal and/or bronchial abnormalities included submucosal nodules (*n* = 20/31, 65%), submucosal calcifications (*n* = 19, 61%), and tracheal stenosis (*n* = 5, 16%) (Fig. 1). Tracheal location was described in 29 cases (94%), and proximal bronchi location in 14 cases (45%). Lung parenchyma abnormalities were described in 16 cases (52%), including consolidation (*n* = 5), emphysema (*n* = 4), localized (*n* = 1) or diffuse (*n* = 3) bronchiectasis and nodules (*n* = 3).

### Bronchoscopic findings

Flexible bronchoscopy was performed in all patients (Table 2), mainly for radiological abnormalities (*n* = 18/35, 50%), among them 9 were linked to TO (tracheal or bronchial nodules or calcifications). TO involved the trachea in 33 cases (92%) and proximal bronchi in 23 cases (64%). The posterior tracheal wall was intact in all patients. Tracheal stenosis was reported in 6 cases (17%) with the severity of stenosis between 20 and 80%. Nodules were scattered in two-thirds of the cases (*n* = 23, 64%).

**Table 2:** Bronchoscopic data

Characteristics	n	%
Number of cases	36	
Indication		
radiological abnormality	18	50
infection	13	36
chronic cough	5	14
hemoptysis	1	3
difficult intubation	2	6
Localization		
trachea	33	92
proximal bronchus	23	64
posterior tracheal wall	0	0
Tracheal stenosis	6	17
Hard consistency of nodules	20	56
Extension classification		
scattered	23	64
diffuse	9	25
confluent	4	11

**Fig. 2:**
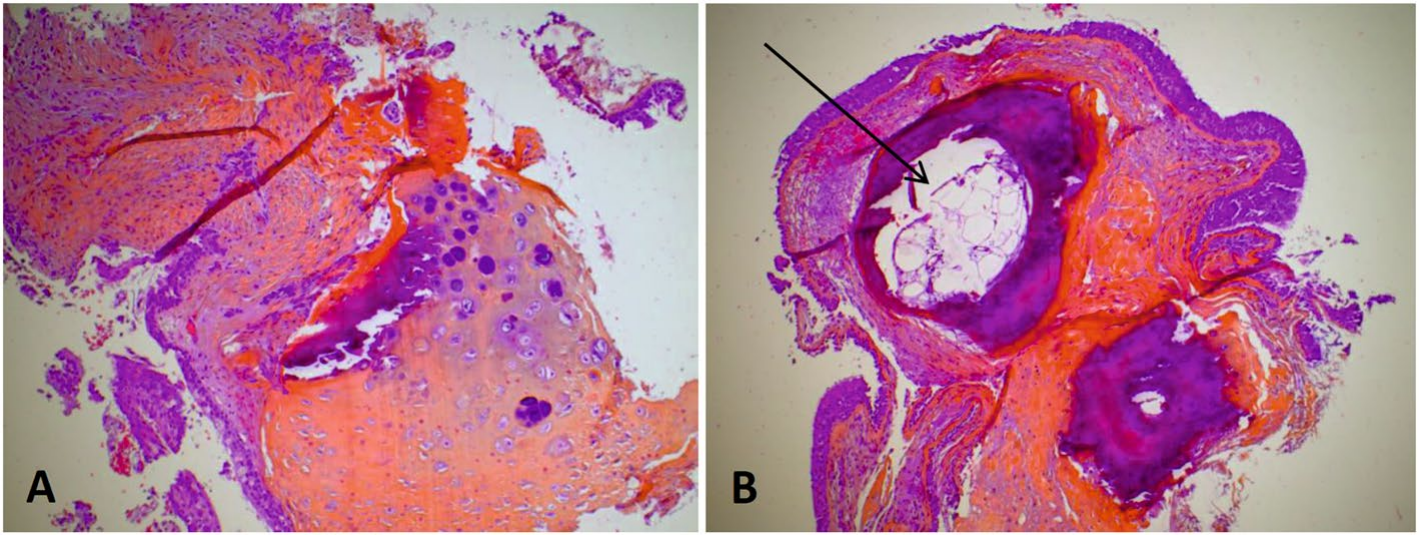
Histopathological features of tracheobronchopathia osteochrondroplastica (bronchial biopsy). HES staining (hematoxylin-eosin saffron), magnification × 400. **A** Voluminous cartilaginous nodule (arrow) in the subepithelial stroma. **B** Two calcified nodules in the subepithelial stroma with one containing bone marrow tissue (arrow)

Biopsies were not performed in 12 cases (43%), mostly because of typical macroscopic TO findings during bronchoscopy. When performed (*n* = 24, 67%), biopsies were made using forceps, and did not induce any local complication except mild hemorrhage in one case. The histological analysis identified a typical aspect of TO in 15 patients (63%), with cartilaginous or bone formations in tracheal/bronchial submucosa (Fig. 2). Non-specific inflammation or squamous metaplasia was described in 5 cases (21%).

Bacteriological analysis from bronchial aspirations at diagnosis was available in 26 cases and did not reveal any significant pathogenic bacteria (i.e. > 10^6^ colony-forming units per mL) in 17 patients (65%). Actinomyces sp. was identified in 2 patients (8%). No *Klebsiella ozaenae* was found.

### Treatment and outcomes

Therapeutic management and follow-up data were available for all 36 patients. No treatment was performed in 31 patients (86%). Interventional bronchoscopy was performed in 3 cases for tracheal stenosis. In two cases mechanical desobstruction through thermocoagulation of large lesions protruding in the tracheal lumen was performed. In the other case exhibiting a bronchoscopically estimated 80% stenosis, a tracheal stent (uncovered, Ultraflex™) was placed but had to be removed because of stent breakage. The patient then underwent laser tracheobronchoplasty to stiffen the posterior tracheal wall before CO2 laser desobstruction [10]. One patient with a history of asthma received a long-term combination of inhaled corticosteroids and bronchodilators. One COPD patient was treated with inhaled bronchodilator and antibiotics for bronchopulmonary infection.

A bronchoscopic follow-up was performed in 7 cases (19%), 9 ± 14 months [1–56] after initial diagnosis. TO bronchoscopic aspect was stable in 6 cases. In the subject treated with a tracheal stent and subsequent laser tracheobronchoplasty, a bronchoscopic improvement of stenosis was noted at 1 month.

## Discussion

Our series of 36 cases of TO is one of the largest reported series [1, 4–6, 11]. Our results identified a typical clinical and bronchoscopic presentation of TO and suggested that no treatment might be required in most cases.

The causes of TO are largely unknown. The diagnosis is usually made during the 5th or 6th decade [1, 4, 11–13], 65 years in our study. TO has been described in children [14, 15], suggesting that TO might occur early in life with a very slow and silent evolution. In our study and others [1, 4, 11], TO occurred in men and women in the same proportion. Compared to other series [1, 4–6, 11], our patients were more frequently smokers (56% vs 18–42%). Occupational exposure to inhaled irritants or pollutants was described in 60% of the cases. Previous studies suggested the role of long-term occupational exposure to dust or irritant gases [1]. The potential link between TO and environmental exposure remains to be elucidated.

In our study, 3 cases were associated with symptomatic GERD, corresponding to the prevalence of GERD in the general population [16]. One patient suffered from atrophic rhinitis, also known as ozena. Atrophic rhinitis is a chronic nasal disease that was suspected to be associated with TO in previous studies [11]. This association has been questioned in more recent studies [4], suggesting a recruitment bias. We did not find any clear link between TO and other respiratory inflammatory diseases (COPD, asthma, chronic rhinitis) or tuberculosis. One case of family history of TO has been previously described [17]. We did not find any familial history of TO in our study.

In our series, a mild to severe airflow obstruction was described in 55% of the cases. The evolution of airflow obstruction is not determined [4, 18]. Long-term follow-up PFT results were not available in our study.

We showed that radiological abnormalities are present in 97% of the cases (82–87% in previous studies [1, 5]) and typically included tracheal and/or bronchial calcifications in chest X-ray and CT-scan submucosal nodules with or without calcifications located in the tracheal and proximal bronchi. CT scan is currently considered the most reliable imaging exam to screen TO [1, 4, 19].

The diagnosis of TO relies on bronchoscopy, which is frequently performed for another medical indication or non-specific symptoms. In our study, 19% of the subjects did not describe any respiratory symptom (5–52% in other series [1, 4, 6],). Our results and others [20–23] identified TO-related symptoms like chronic cough, dyspnea on exertion, hemoptysis, and dysphonia. One patient in our study underwent bronchoscopy to explore difficult intubation occurring during abdominal surgery. Similar cases have been previously published [24–26]. TO should be considered in case of difficult intubation and attention should be paid to the CT scan regarding tracheal abnormalities.

The bronchoscopic aspect of TO is characterized by the presence of numerous cartilaginous and/or bony nodules on the submucosa of the tracheobronchial tree, protruding in the lumen and typically sparing the posterior membranous wall [5]. Several descriptions have been made regarding the typical aspect of TO on bronchoscopy, depending on the severity of obstruction: cobblestone, stalactitic cave, mountainscape, or rock garden [5]. Biopsies of these lesions can be difficult, mainly because of the hard consistency of nodules, and usually do not induce important bleeding [27]. Another characteristic is the grinding of the bronchoscope under the lesions [28]. In our study, lesions were predominantly scattered, involving the trachea and proximal bronchi in most of the cases, and spared the posterior wall in all cases.

TO can induce tracheal stenosis (10–27% [1, 4, 5, 11], 17% in our study), sometimes important [29, 30], and then require specific endoscopic management (4–5% in the previous series, 8% in our study). Another complication of TO is bronchopulmonary infections [31], probably linked to mucociliary system failure. *Klebsiella ozaenae* is a Gram-negative organism colonizing the oral and nasopharyngeal mucosa. K. ozaenea has been thought to be involved in TO physiopathology through the alteration of the mucociliary system and induction of squamous metaplasia [32]. We did not identify any *K. ozaenae* in bronchial aspiration samples in our study.

The need for histopathologic proof of TO is controversial; some authors consider that typical findings on bronchoscopy are sufficient to diagnose TO [33]; others consider it necessary to exclude differential diagnoses such as tracheal amyloidosis, polychondritis, or papillomatosis [1, 34]. In our study, biopsies were performed in 42% of the patients, less than 64–70% described in other series. Typical histologic findings of TO, i.e. cartilaginous or bone formations in tracheal/bronchial submucosa can be frequently observed (63% in our study).

There are currently no guidelines for the management of TO. It usually depends on the severity of proximal airway obstruction. In our retrospective case series, TO therapeutic management was based on the physician’s experience and bronchoscopy habits. Different treatments have been described, including inhaled corticosteroids [3], bronchoscopic management or surgery [35] for the most severe cases with tracheal stenosis. In our study, bronchoscopic management was performed in 3 cases with tracheal stenosis. One subject has been treated with corticosteroids that have been described as improving symptoms, radiologic and bronchoscopic findings in some cases [1]. Two patients received bronchodilator treatment for either asthma, in combination with inhaled corticosteroids, or COPD with no purpose of any impact of bronchodilator treatment on TO. Further studies should analyze the interest of such long-term inhaled treatment in the evolution of TO.

TO usually has a benign course [36] but can sometimes be evolutive and life-threatening [37, 38]. In our study, 7 patients underwent a bronchoscopic reevaluation, that identified stability of TO in all cases.

The pathophysiology of TO remains unclear. Several assumptions have been made: one hypothesis suggested the role of ecchondrosis and exostosis from the lateral tracheal rings [39, 40]. Another hypothesis suggested metaplasia followed by ossification of the connective tissues [4]. The role of Bone Morphogenic Protein 2 (BMP-2) and Transforming Growth Factor beta-1 (TGF beta-1) in nodular formations has been suggested [41]. As described in other studies [1], a potential tracheal and bronchial chronic inflammation, associated with COPD, smoking, bronchopulmonary infections, or occupational exposure was described in most of our cases. Based on these elements, chronic inflammation could play a role in the development of TO. A very recent genome-wide study analyzing the whole genome expression and epigenetics of tracheal-bronchial basal cells obtained from TO and non-TO subjects identified a role of these cells in epithelial metaplasia and mesenchymal osteo-chondrogenesis, highlighting a malfunction of airway stem cells inducing neo-osteogenesis in TO [42].

Our study has some limitations, including its retrospective design, and the limited number of cases, although it is one of the largest reported to date. Given the lack of guidelines currently available for the diagnosis and therapeutic management of TO, we report here individual management based on the physician’s experience and bronchoscopy habits, especially regarding the indication for biopsies or bronchoscopic management. Despite those limitations, our study provides a clear picture of the clinical and bronchoscopic presentation of TO and reports the experience of TO management by bronchoscopy experts, which could be useful to less experimented physicians in such a rare condition.

## Conclusions

In summary, the diagnosis of TO relies on typical bronchoscopic features and can be suspected based on CT-scan findings. Given its low consequences in terms of symptoms, lung function, and evolution, no treatment is usually required apart from rare severe tracheal stenosis. Despite some recent advances in the pathophysiological mechanisms, other studies are needed to investigate long-term outcomes and therapeutic management of TO.

## Data Availability

All data generated or analysed during this study are included in this published article.

## Abbreviations

TO: Tracheobronchopathia osteochondroplastica
GETIF: Groupe d’Endoscopie Thoracique et Interventionnelle Francophone
FEV1: Forced expiratory volume in the first second
FVC: Forced vital capacity
RV: Residual volume
TLC: Total lung capacity
CT: Computed tomography
COPD: Chronic obstructive pulmonary disease
GERD: Gastroesophageal reflux disease
PFT: Pulmonary function tests
BMP-2: Bone morphogenic protein 2
TGF-β1: Transforming growth factor β1
